# Practical Observations on the Treatment and Causes of the Dropsy of the Brain

**Published:** 1791

**Authors:** Thomas Percival

**Affiliations:** President of the Literary and Philosophical Society of Manchester; Member of the Royal Medical Society at Paris; of the Royal Society of Agriculture at Lyons; of the Medical Society of Aix in Provence; of the Philosophical Society at Philadelphia; and of the American Academy of Arts and Sciences, &c.


XIII. *Practical Obfervations on the Treatment and Caufes of the Dropfy of the Brain. By* Thomas Percival, *M. D. F. R. S. and S. A. Lond.; F. R. S. and R. M. S. Edinb.; Prefident of the Literary and Philofophical Society of Manchefter; Member of the Royal Medical Society at Paris; of the Royal Society of Agriculture at Lyons; of the Medical Society of Aix in Provence; of the Philofophical Society at Philadelphia, and of the American Academy of Arts and Sciences, &c.*

THE fafety and efficacy of mercury in the hydrocephalus internus has been fully eftablifhed, by the experience of various medical practitioners, in this and other countries fince

ſince the year 1777, when the public was firſt informed of its ſucceſsful adminiſtration *; and though it is far from being a certain remedy, yet the almoſt conſtant fatality of this diſorder, under every antecedent method of cure, renders it a valuable and important acquiſition to the healing art. But in the recital of one of the earlieſt caſes in which it was employed I have perhaps too much diſparaged former modes of treatment, and too haſtily declared my ſole and excluſive truſt in the internal and external uſe of mercury; for there are ſeveral medicinal aids, which, however inſufficient in themſelves to conquer this formidable diſeaſe, may contribute to ſo happy an event, by mitigating pain and ſpaſm, by promoting abſorption, and by increaſing the ſerous diſcharges of the body. With theſe views I now generally preſcribe either opium, muſk, ſalt of hartſhorn, flowers of zinc, ſquills, or bliſters, in conjunction with the mercurial courſe, with which they perfectly coincide. The preference to be given to one or other of theſe remedies the circumſtances of the

* See Medical and Philoſ. Commentaries, Vol. V. p. 174, Vol. VI. p. 219; Medical Obſervations and Inquiries, Vol. VI. art. vi. by Dr. Dobſon, art. viii. by Dr. Haygarth.

the caſe will ſufficiently indicate; and it would be a falſe and unjuſtifiable ſacrifice to ſimplicity of practice not to avail ourſelves, in the treatment of ſo dreadful a malady, of ſubordinate means, which may prove auxiliary, and cannot counteract the ſalutary powers of what merits our chief reliance. In purſuing this enlarged plan, I have experienced fewer diſappointments than formerly, and have derived ſatisfaction under it, from the conſciouſneſs of no neglect or omiſſion. When the pains are very acute, opiates, in large and repeated doſes, are eſſential to the cure; but if the patient be in a ſtate of *coma*, they are obviouſly improper, and muſk, combined with ſalt of hartſhorn, ſhould be freely adminiſtered. Under every circumſtance of the diſeaſe bliſters are expedient; and the application of them ſhould be renewed as often as can be done without exciting ſtrangury. I have tried fox glove in but few caſes of hydrocephalus. In one, which was of long continuance, and terminated fatally, this remedy produced extreme debility, various diſtreſſing nervous ſymptoms, and was attended with no beneficial operation. Mercurials, and other active means, had alſo been tried in vain; and all hopes of relief being given up, the child was ſent into the country, where, though totally blind, he was not without enjoyment; and retained his faculties in a perfect ſtate till death, notwithſtanding it was found, on opening the head, that the right hemiſphere of the brain was entirely diſſolved, the corpora ſtriata deſtroyed, and the left lateral ventricle contained twelve ounces of water. In another caſe, which lately occurred, fox glove was adminiſtered, with opium and calomel, according to the following formula:

℞. Pulv. digital. purpur.

Opii colati,

Calomel. pp$^{ti}$, aa g$^{r}$ j. M.

F. Pilulæ ij. 4$^{tis}$ horis ſumendæ.

After the ſecond doſe of theſe pills, the patient, who was about twelve years of age, fell into a ſound ſleep, which continued ſix or eight hours. She awaked, in a great meaſure, free from pain, highly refreſhed, and capable of viewing the light. Her head had ſweated profuſely, a large flow of urine had taken place, and from this period the commencement of recovery was clearly to be dated. But I am inclined to aſcribe the ſalutary change rather to the the opium than to the fox glove, and to the opium only as auxiliary to the powerful action of the mercury, which had been previoufly and very largely adminiftered in the way of unction; and I am ftrengthened in this conclufion by the antecedent effects of mercury in the fame patient, under the direction of Mr. Henry, before I was confulted; for confiderable relief had been obtained by it, though afterwards fhe fuffered a relapfe; and the courfe was renewed by my advice, with the additional remedies above mentioned.

I have examined various hiftories of hydrocephalus, related in different medical journals, fince the adoption of mercury in the treatment of it, and of twenty-fix cafes, noted indifcriminately in the courfe of my inquiries, I find that eleven recovered, and fifteen died. Of the recoveries, mercury was employed in feven of the inftances, and other remedies in the remaining four. Of the deaths, mercurials were employed in four cafes, and other remedies in eleven. Thefe facts, drawn from authentic records *, afford a ftriking proof of the fuperior advan-

* Medical Obfervations and Inquiries; Dr. Duncan's Medical Commentaries; London Medical Journal; Memoirs of advantages of the prefent method of practice in a difeafe heretofore deemed fo fatal, and my own experience confirms the conclufion ; for in the above references no cafes are included which fell under my own infpection.

A profufe perfpiration of the head is not an unfrequent effect of the exhibition of mercury; and it fhould be encouraged by wrapping the head in flannel, for it fometimes affords fpeedy relief, of which I fhall relate, from my notes, the following inftance :—October 23, 1784, I vifited Mafter E., a child of eighteen months old, who laboured under the hydrocephalus internus : his eyes were diftorted, and in every pofition, without the power of feeing : his pulfe beat one hundred and fixty ftrokes in a minute : he fhewed figns of great pain in his head, and frequently rocked it on the pillow. The right arm and leg were motionlefs. The bregma was foft and enlarged. I directed a blifter, the *unguent. hydrarg. fort.*, and fmall dofes of mercury internally. In twenty-four hours a moft profufe fweating of the head came on. The pain and diftortion of the eyes were confideraof the Medical Society of London ; Dr. Donald Monro's Letters and Effays.

bly bly abated. Little inflammation or ſerous diſcharge had been produced by the bliſter.—October 25th, the power of motion was reſtored to his paralytic arm and leg, and he ſaw objects diſtinctly, as appeared by his catching at my watch when held before him.—On the 27th he relapſed into his former ſtate.—October the 30th he died; but permiſſion to examine the head could not be obtained.

The good effects of mercury in hydrocephalus internus are independent of ſalivation; and it is truly aſtoniſhing that very large quantities of the *unguent. hydrarg.* may be uſed in infancy and childhood without affecting the gums, notwithſtanding the prediſpoſition to a flux of ſaliva at a period of life incident to dentition. Between the 8th of February and the 7th of April, 1786, a child, under one year of age, received, by ſucceſſive frictions, four ounces, ſix drachms, and two ſcruples of the ſtronger mercurial ointment. One ſcruple was adminiſtered each time: the operation took up more than half an hour, and the part to which the ointment was applied was always previouſly bathed with warm water, precautions adapted to ſecure the full abſorption of the mercury. Thirty-ſeven grains of calomel were alſo given at proper intervals, and in ſixteen doſes, during the ſame period. The child recovered without ſymptoms of ſalivation, and has ever ſince remained free from any painful affection of the head.

In the proſecution of the mercurial courſe it is neceſſary to guard againſt laxity in the bowels, or this remedy will be carried out of the ſyſtem, and produce no ſalutary effect. Coſtiveneſs, however, is not to be permitted, leſt it increaſe ſickneſs, fever, and pain: clyſters, therefore, of milk, oil and ſalt, ſhould be injected as often as they may be deemed expedient.

An acceleration of growth, to an extraordinary degree, is frequently obſerved after the dropſy of the brain has been ſubdued by mercury. In one caſe, which fell under my own direction in 1784, a young lady, nine or ten years of age, of a noble family in this county, increaſed two inches in ſtature within the ſpace of four months ſucceeding her recovery.

The comparative frequency of the hydrocephalus at different periods of life, the prediſpoſition of each ſex to it, and the duration of the diſeaſe, as deducible from the records of the twenty-ſix caſes to which I have already referred, are noted in the following table:

Age.

*Age.*

| | | | | |
|---|---|---|---|---|
| From birth to one year | — | — | | 2 |
| —— one to two years | — | — | | 1 |
| —— two to five | — | — | — | 8 |
| —— five to ten | — | — | — | 6 |
| —— ten to twenty | — | — | — | 5 |
| —— twenty to thirty | — | — | | 4 |
| | | | | — |
| | | | | 26 |

| *Sex.* | | | *Event.* | | |
|---|---|---|---|---|---|
| Males | — | 14 | Died | — | 15 |
| Females | — | 12 | Recovered | — | 11 |

*Duration* *.

| | | | |
|---|---|---|---|
| Two weeks and under | — | — | 7 |
| From two weeks to three, inclusive, | | — | 6 |
| —— three weeks to four, ———, | | — | 4 |
| —— one month to six weeks, ——, | | — | 1 |
| Three months, with intermission, | | — | 1 |

This table is not sufficiently comprehensive to furnish any decisive conclusions; and it was my intention to have enlarged it, if leisure had

* The duration, I believe, is only stated in nineteen of the twenty-six cases.

permitted, by a review of the numerous cafes which have occurred in my own practice. Several at this inftant prefent themfelves to my recollection, which were of very long duration. The child mentioned above, to whom fox glove was given with injurious effect, fubfifted under the difeafe fixteen months. The malady came on by almoft infenfible degrees, and was attended with very little pain: his head was large, but neither the bregma nor the futures were open: his eyefight failed, and a complete amaurofis fucceeded. The left fide became paralytic; and in this melancholy fituation he continued till a fhort and flight attack of fever put an end to his exiftence.—Another cafe, about which I was confulted, I fhall relate more at length, as it was attended with many curious and interefting circumftances. A lady, aged twenty-five years, of a healthy conftitution, and of a florid complexion, was delivered of her third child in July, 1786. During the latter months of pregnancy fhe was fubject to frequent head-achs, attended with great coldnefs of the legs and feet. The head-achs abated after delivery; but in the fucceeding month of October they recurred periodically every morning, and were generally alleviated during the courſe of the day. At this time ſhe ſuffered extremely from habitual coſtiveneſs. In December her oldeſt child, whilſt ſitting on her knee, was ſeized with a convulſion, which occaſioned ſuch terror and agitation in the mind of the mother as to aggravate, to a very great degree, the pains of her head. Bliſters, an emetic, and other means were directed, without any material or laſting relief. In February, 1787, a ſlight ſhaking of the head came on; and a phyſician in London, deſervedly held in high eſtimation, was conſulted, who delivered it as his opinion that the diſeaſe was an extravaſation of water in the brain. At this period the pupils of the eyes were greatly dilated, and the ſight ſo much affected, that ſhe was incapable of reading. Her ſpeech alſo was ſcarcely intelligible. In April application was made to another very eminent phyſician, who directed an iſſue between the ſhoulders capable of holding twenty-four peas, and afterwards adviſed the mercurial ointment, which was employed in the quantity of one drachm every night during the ſpace of a month. A profuſe ſalivation ſucceeded, but with little or no benefit. Electricity, the Bath waters, frequent emetics, ſtimulating cataplaſms to the head, &c. were equally equally inefficacious. February the 14th, 1788, I received a ſtatement of the caſe. The lady had then for ſome time declined all medical aſſiſtance; but ſhe ſuffered under an almoſt general paralyſis, and was incapable either of dreſſing or of feeding herſelf. Her head was ſometimes totally free from pain during the ſpace of a day or two, but always in ſo ſhaking a ſtate, that ſhe was obliged to uſe a ſteel ſupporter. The muſcles of the jaws were likewiſe ſo relaxed, that manducation was performed with great difficulty. In this long-protracted caſe there is reaſon to ſuppoſe that a ſerous effuſion ſubſiſted both in the ventricles and between the membranes of the brain; and it is not unlikely that the ſpinal marrow alſo was affected by a ſimilar compreſſion. Under ſuch circumſtances, and after the trial of ſo many active remedies in vain, directed by phyſicians of diſtinguiſhed ſkill and judgment, I felt a peculiar diffidence in offering any encouragement for the uſe of farther means. But when a great and good end is in view, the poſſibility of attainment is not only a juſtifying motive of exertion, but even renders it a duty; and experience evinces that ſucceſs ſometimes crowns our endeavours when there are no probable grounds of expectation of it. I therefore recommended that the ftrength of the patient fhould be fupported by a cordial and nutritive diet; by refidence in a dry and temperate atmofphere; and by gentle exercife, either natural or artificial. Of the latter kind, friction of the abdomen and of the fpine, with new flannel, every night and morning, was particularly advifed. As the iffue in the back appeared to be too wafting a drain in fo advanced a period of the diforder, I hinted the propriety either of healing it or of dreffing it only with one pea. The vapour bath was propofed to the lady, which, by warming the whole habit, might give energy to the nervous and lymphatic fyftems. As the mercurial courfe had been tried without benefit, it could not be deemed expedient to renew it to any extent: but I conceived that fmall quantities of the *unguent. hydrargyri* might be rubbed, with advantage, into the fpine every night at bed time. If this were objected to, I mentioned the ufe of a volatile embrocation as a fubftitute. Sternutatories having fometimes proved beneficial in hydrocephalic affections, I therefore fuggefted the ufe of them in the prefent cafe; and recommended the trial of mufk, æther, and the calx of of zinc. If an adequate degree of ſtrength to bear the fatigues of a ſea voyage were recovered by theſe or other means, I urged the attempt of it, at a proper ſeaſon of the year, as likely to produce a ſalutary revolution in the whole ſyſtem.

Such was the advice I gave in this intereſting and affecting caſe; and I regret that no other information has been communicated to me, of the reſult of it, than that the lady received conſolation and benefit from the plan propoſed to her trial.

Our imperfect knowledge of the ſtructure of the brain, and of the diverſified energy of the nerves in their origin, progreſs, and termination, neceſſarily involves the diſorders of the head in a peculiar degree of uncertainty; and it is often extremely difficult to diſcriminate even between the ſympathetic and idiopathic affections of that important organ. It cannot, therefore, be ſurpriſing that the cauſes of hydrocephalus have not hitherto been aſcertained with any degree of accuracy or preciſion. The light which diſſections afford is obtained only at the cloſe of the malady; and the ſtate of the encephalon may have undergone conſiderable changes, either by the operation of nature, or by by the action of the medicines employed. A medical friend informs me that he lately aſſiſted at the diſſection of a child whoſe death was the conſequence of a convulſive fit. The child had been indiſpoſed about two months with frequent pains of the head. Worms had been ſuſpected; but anthelmintic remedies afforded no relief. The veſſels of the brain were found to be uncommonly turgid, and the ventricles contained about double or treble the ordinary quantity of ſerum. In this caſe I apprehend the turgeſcence of the veſſels was the effect, and not the cauſe, of the convulſions; for the reflux of the blood from the head to the heart being obſtructed during the fit, in which I believe the patient expired, the vaſcular diſtention muſt have been permanent. The redneſs and even blackneſs of the face, which takes place in convulſions, affords ſufficient proof of ſanguineous accumulation.

That the diſorder under enquiry originates ſometimes in inflammation, I can entertain no doubt, from ſeveral caſes which have occurred within the circle of my obſervation; one of which I ſhall here briefly relate. Miſs ——, a robuſt young lady, at a boarding-ſchool in Mancheſter, after dancing very violently, went out to the pump for a glaſs of cold water, which ſhe eagerly and haſtily drank. In a few minutes ſhe ran towards the wall, and leaning againſt it, cried out, oh my head! From this inſtant the pain never ceaſed; and becoming more and more acute, was ſucceeded by all the train of afflictive ſymptoms which characterize the hydrocephalus. At the end of three weeks ſhe died, and I was preſent at the opening of the head. The brain was found to be in a ſtate of inflammation; the veſſels were extremely turgid; and the venticles contained at leaſt ſeven ounces of water.

But I have reaſon to believe that hydrocephalus moſt frequently ariſes from glandular obſtruction, and either local or general plenitude. A family, with which I have been long and intimately connected, has repeatedly ſuffered from this formidable diſeaſe, as diſſections have manifeſted. But in the following inſtance, the fatal event took place before the extravaſation of water, which, from the ſtate of the brain, and the conſtitutional prediſpoſition, would probably have ſucceeded, if the diſeaſe had been of longer continuance. Maſter ——, a fine healthy boy, aged fifteen weeks, who fed heartily and grew very faſt after he was weaned; though though regular in his ſtools and well exerciſed, was ſeized with convulſions, May 11th, 1772. He had been obſerved, a day or two preceding this attack, to be averſe to motion, and as there appeared to be no ſpecific ſymptoms of ſmall-pox or dentition, the diſeaſe was aſcribed to general plenitude. Gentle evacuations were therefore directed; bliſters applied; and anodyne and antiſpaſmodic remedies adminiſtered. By theſe means the fits gradually abated in frequency and violence. May 13th, the convulſions entirely ceaſed, and every ſymptom ſeemed to be favourable: but in the evening, his pulſe intermitted, his reſpiration became laborious, and ſometimes ſuſpended, and his countenance was ſunk and ghaſtly. By ſinapiſms, bliſters, and cordials, the vital powers were again rouſed: but the next evening he relapſed, and died early the ſucceeding morning. His body was opened. The viſcera of the abdomen and thorax were ſound, the meſentery excepted, the glands of which were much obſtructed and enlarged. The veſſels of the brain were turgid, but not inflamed; and no water was yet effuſed, either between the membranes or into the ventricles.

In a chronic affection of the head, of many years years continuance, the patient informed me, that to hide certain marks left by glandular tumours in his neck, he uſed, for a long time, a ſtrong and broad ſtiffening under his neckcloth; and frequently tied it ſo tight, as to occaſion pains of the head, fluſhings of the face, and giddineſs. This practice he purſued till the commencement of the melancholy diſorder, about which he conſulted me; and was perſuaded that it greatly contributed to bring it on. The ſymptoms of the caſe were acute pains of the head; a vertigo in ſtooping; a ſenſe of fainting, when the head was ſuddenly thrown back; a ſtrabiſmus, and protruſion of one of the eyes; a paralytic debility of the whole frame; and a conſiderable diminution of mental energy. By the continued uſe of tonics and ſtimulants; by a cordial regimen; and by drinking goats-whey on the mountains in Wales, this gentleman's health is, in a tolerable degree, reſtored. Amongſt the ſtimulants employed, the mezereon root, mercury, and bliſters are comprehended. In this ſingular caſe, the internal veſſels of the head were probably at firſt over diſtended; and ſome degree of ſerous extravaſation permanently enſued. But the complaint was ſo gradually produced, that the vital actions ſuſtained a laſting injury injury from turgefcence of the brain, without any concomitant inflammation.

In reviewing fome notes on Hydrocephalus, which I made many years ago, I find that of twenty-two cafes enumerated, eleven were known to be ftrumous; three were fufpected to be fuch; and four were accompanied with an enlargement of the head, probably from a rickety conftitution. So that in eighteen of thefe cafes, the inflammatory diathefis was not likely to prevail. But fuch fubjects, from the laxity of their fibres, muft have been incidental to general or partial plenitude; to glandular obftructions, and to a feeble action of the lymphatic fyftem. And thefe circumftances peculiarly difpofe to an effufion of water in the brain; becaufe that organ is fo copioufly fupplied with veffels of every order. For anatomifts have eftimated that one tenth of the whole mafs of blood circulates within it, although the weight of the encephalon does not exceed one fortieth part of the whole body. The following cafe affords fome confirmation of this reafoning. Mafter ——, the fon of a ftrumous parent, was born with a large head. Being a lufty child and growing well, the difproportion became lefs obfervable within the fpace of two years.

He was then attacked with a malignant itch, the cure of which proved very obſtinate; and when the eruption gave way, its receſſion was too ſudden. The child's appetite abated, and his fleſh, ſtrength, and ſpirits were much impaired. He was ſent into the country, where he ſeemed to be recruited. But about the end of May, he ſickened, grew languid, averſe to food, dull, watchful and yet drowſy. His countenance had that peculiar caſt, which led me to ſuſpect an effuſion of water in the brain, though his pulſe was not much changed, nor his eyes otherwiſe affected than by the loſs of their uſual brightneſs. June 2d. The pupils became dilated, and the pulſe was ſometimes ſlow and ſometimes quick. The child ſeemed to feel uneaſineſs in the head, but gave no ſigns of acute pain. June 3d. A complete amauroſis took place, with occaſional ſquinting and frequent convulſive ſtretchings of the limbs. The pulſe continued to be irregular, and the urine was voided in large quantities. No ſymptom of acute pain occurring, I examined the head attentively, but did not perceive either any opening of the ſutures, or ſoftneſs of the bregma. The mother informed me that the child's head had been gradually augmenting in

ſize,

ſize, during the laſt three months, without a proportionate growth of the reſt of the body, inſomuch that ſhe had been obliged to make for him new and large night-caps.

Practical writers have related many caſes of dropſical *metaſtaſis*. I have ſeen an affection of the brain, which appeared to be hydrocephalic, and probably originated in inflammation, ſuddenly and completely relieved by the attack of an acute pain in the ſide, which terminated in a fatal abſceſs and hydrothorax. When an inciſion was made through the cartilage of the third rib, a quantity of water flowed out of the cheſt. The ſternum being removed, evident marks of inflammation were diſcovered; the lungs adhered ſtrongly on every ſide to the pleura, and were covered with a purulent matter. An open abſceſs was found in the poſterior lobe of the left ſide, the rupture of which, in all likelihood, occaſioned the ſudden death of the patient. Not being preſent at this diſſection, I had afterwards to regret that the ſurgeon omitted to examine the ſtate of the brain.

Whether the following caſe, with which I ſhall conclude theſe obſervations, is to be aſcribed to *metaſtaſis*, I leave to the deciſion of the reader. Mr. C.'s daughter, aged nine years, after labouring under the ſymptoms of phthiſis pulmonalis four months, was affected with unuſual pains in her head, which increaſed rapidly to ſuch a degree, as to occaſion frequent ſcreamings. The cough, that had before been extremely violent, and attended with ſtitches in the breaſt, now abated; and in a few days ceaſed almoſt intirely. The pupils of the eyes became dilated; a ſtrabiſmus enſued; and in about a week, death put a period to her agonies.

P. S. The foregoing practical obſervations have been drawn up from my notes, or from reflection, without conſulting, at the time, any books on the ſubject. But ſince theſe were written, I have peruſed, with attention, a valuable and very intereſting work, lately publiſhed by Dr. Quin, on the dropſy of the brain. The view which he has given of this diſeaſe appears to be, in general, judicious and accurate: yet I am inclined to aſcribe leſs than he does to inflammation; and conſequently ſhould recommend much caution in the uſe of blood letting, even in the firſt ſtage. For the veſſels of the brain ſeem quickly to loſe their tone by diſtention; and great torpor and debility of the whole ſyſtem ſucceed. If depletion, therefore, be indicated dicated, it will be beſt accompliſhed by a ſtimulating mercurial purgative, and by the application of a large bliſter to the head. For Dr. Quin is miſtaken in ſuppoſing, that the mercurial unction was ever directed to that part of the body, either by Dr. Dobſon or myſelf. He has fallen likewiſe into another error, in ſtating that the firſt patient cured by Dr. Dobſon, of Hydrocephalus, was his own child, and that the fortunate event depended on the opportunity that this afforded of the moſt early attention. This, indeed, was the caſe, with reſpect to one of the early hiſtories, which I have related; and I acknowledge the juſtice of the Doctor's concluſion concerning it.

*Mancheſter,*

May 20, 1791.

